# Long-range diffusion of bound Ca^2+^: time-dependent WKBJ solutions in one- and two-dimensional imaging

**DOI:** 10.3389/fncel.2026.1925214

**Published:** 2026-10-05

**Authors:** Sergej L. Mironov

**Affiliations:** Institute of Neuro- and Sensory Physiology, Georg-August-University, Göttingen, Germany

**Keywords:** analytical solutions, buffer motility, cytoplasmic calcium profiles, L-type Ca2+ channels, propagating calcium signals, synuclein channels, WKBJ

## Abstract

**Introduction:**

Reaction–diffusion (RD) systems play a fundamental role in numerous biochemical and biophysical processes. Here, we present a novel analytical framework for solving RD equations by applying the Wentzel–Kramers–Brillouin–Jeffreys (WKBJ) formalism to Ca²⁺ buffering. Previous models have primarily focused on stationary Ca²⁺ nanodomains while neglecting diffusion and saturation of intracellular Ca²⁺ buffers and sensors.

**Results:**

We derive analytical solutions using WKBJ ansatz and demonstrate that sustained Ca²⁺ source produces continuously expanding distributions of free Ca²⁺, whereas the concentration of Ca²⁺-bound buffers develops at much slower timescale. These predictions are supported experimentally by measurements of one-dimensional fluorescence profiles produced by single-channel activity and two-dimensional profiles generated by whole-cell Ca²⁺ currents.

**Discussion:**

The analytical framework developed here extends Michaelis–Menten-type kinetics to reaction–diffusion systems and may therefore be broadly applicable to biochemical and biophysical processes in which diffusion cannot be neglected.

## 1 Introduction

The Michaelis and Menten (1913) equation and its associated quasi-steady-state approximation (Segel and Slemrod, 1989; Bersani et al., 2015; Choi et al., 2017) have served for more than a century as fundamental tools for describing biochemical and biophysical reactions. Despite numerous refinements, these formulations generally describe either spatially homogeneous systems or highly localized reactions in which diffusion is neglected.

For intracellular Ca^2+^ signaling, Wagner and Keizer (1994) incorporated Michaelis–Menten kinetics, also known as the rapid buffer approximation (RBA) for Ca^2+^ buffering, into a reaction–diffusion (RD) framework and derived a non-linear equation for total Ca^2+^ concentration, which they analyzed numerically and under several limiting approximations. Mironova and Mironov (2008) found that total Ca^2+^ can be well described using the logarithmic approximation and derived analytical formulas for transient free Ca^2+^ dynamics. They also emphasized that Ca^2+^ is transported predominantly in its bound form within the cytoplasm.

Many endogenous Ca^2+^ buffers diffuse slowly or are effectively immobilized (Zhou and Neher, 1993; Schwaller, 2010). Mironov (2023a) analyzed the case of irreversible Ca^2+^ binding and derived analytical expressions that predicted a localized accumulation of free Ca^2+^ around a single channel. Ca^2+^-bound buffer species were found to propagate away from it much faster. Since endogenous buffers and sensors are usually large proteins with limited mobility, the propagation of bound Ca^2+^ may represent an efficient mechanism for long-range intracellular signaling. In this view, Ca^2+^-bound molecules, rather than free Ca^2+^ itself, may constitute the principal carriers of information between distant intracellular sites.

At first sight, the hypothesis may appear inconsistent with the widely accepted concept of stationary Ca^2+^ nanodomains surrounding single channels (Neher, 1986; Eisner et al., 2023). However, these perspectives are not mutually exclusive, as discussed below.

In the present study, we revisit Michaelis–Menten-type kinetics by explicitly incorporating diffusion and derive analytical solutions using the Liouville–Green (LG) transformation, commonly known in quantum mechanics as the Wentzel–Kramers–Brillouin–Jeffreys (WKBJ) approximation. Although WKBJ methods are routinely applied to stationary problems, their use in non-stationary RD systems has remained limited (Krause et al., 2020).

To maintain the accessibility of the main text, all mathematical derivations are presented in Sections 5.1 and 5.2. We demonstrate that the leading-order term of the LG/WKBJ expansion accurately reproduces multidimensional diffusion with and without reactions and provides simple analytical expressions for Ca^2+^ buffering. Experimental measurements obtained in one-dimensional single-channel recordings and two-dimensional neuronal preparations support the theoretical predictions.

Overall, our findings suggest that Ca^2+^-bound species can propagate more rapidly and over greater distances than localized free Ca^2+^ signals, potentially influencing the timing and spatial organization of intracellular signaling. In addition to Ca^2+^ dynamics, the analytical framework developed here offers a straightforward approach for incorporating diffusion into Michaelis–Menten-type reaction systems.

## 2 Methods

### 2.1 Ethical approval

All animal experiments were approved by the Lower Saxony State Office for Consumer Protection and Food Safety and conducted in accordance with institutional and national guidelines.

### 2.2 Cell preparation

Cultured hippocampal neurons and chick dorsal root ganglion (DRG) neurons were prepared as previously described (Mironov, 1990; Mironov et al., 1993, respectively). DRG neurons were used 6–12 h after plating, when they had fully adhered to the substrate and exhibited a flattened circular morphology with a thickness of approximately 2–3 μm. At this time in culture, spontaneous synaptic activity was absent, minimizing contributions from neuronal network activity.

### 2.3 Solutions

In single-channel measurements, the solutions contained 30 mM Tris buffer (pH 7.4) together with either 154 mM NaCl (pipette) or 88 mM CaCl₂ (bath). Fluo-4 and Fluo-4 dextran (30 kDa) were used at final concentrations of 0.2 mM. In whole-cell recordings, the intrapipette solution contained CsMeSO4 (92 mM), CsCl (43 mM), TEA-Cl (5 mM), MgCl2 (1 mM), HEPES (10 mM), ATP (4 mM), and GTP (0.4 mM); a pH of 7.4 (titrated with CsOH) was used. The indicators were added at 200 μM.

### 2.4 Patch-clamp recording and imaging

Electrophysiological recordings and fluorescence imaging were performed as previously described. α-Synuclein channels were incorporated into inside-out membrane patches excised from the cultured hippocampal neurons (Mironov, 2015). Opening of individual channels generated outward Ca^2+^ currents that produced localized increases in Ca^2+^-bound fluorescence within the recording pipette.

In whole-cell recordings, voltage steps were applied from −60 to −10 mV and evoked Ca^2+^ currents that were predominantly carried by L-type Ca^2+^ channels.

Fluorescence imaging was performed using total internal reflection fluorescence microscopy (TIRF) combined with high-speed electron-multiplying charge-coupled device (EMCCD) acquisition. In whole-cell recordings, the experimental protocol—control, BayK 8044, and nifedipine (see Methods)—was applied when the L-type current showed no significant rundown within the first 5 min.

For imaging, an upright microscope (Axioscope 2, Zeiss) with a 63 × objective lens (numerical aperture, 1.4) was used. A laser beam delivered 488-nm light from below through a prism (TIRF Labs, Inc., Cary, NC) at an angle appropriate for TIRF. The 525-nm emission passed through a dichroic mirror was filtered at 535 nm. Images were captured using a cooled iXON Ultra 888 EMCCD camera (ANDOR, Oxford Instruments) operated with ANDOR software. In the one-dimensional (1D) experiments, line-scan mode (512 × 2 pixels at 12-bit resolution) was used, corresponding to an acquisition time of 0.1 ms in Crop mode. In whole-cell recordings, the recorded area was 128 × 128 pixels, and the acquisition time was 1.5 ms. Fluo-4 fluorescence increases >30-fold after Ca^2+^ binding that minimized out-of-focus effects. The lateral resolution, measured using 0.1-μm TetraSpeck beads (ThermoFisher, Germar, Germany), yielded a lateral point-spread function of 0.18 μm. The TIRF depth was approximately 200 nm, allowing the flattened DRG cells in culture to be considered planar objects and the results obtained for Ca^2+^ diffusion in circular geometry to be applied. Image analysis was performed offline using MetaMorph software. Images were background-subtracted and median-filtered before analysis. Fluorescence changes are reported as relative changes (ΔF/F₀).

### 2.5 Statistics

Each experiment was repeated in at least four cells obtained from different preparations. Data are presented as mean ± SD. Statistical significance was assessed using paired t-tests where appropriate, with significance accepted at *p* < 0.05.

### 2.6 Reaction–diffusion model

Ca^2+^ diffusion in the presence of a single buffer was described by coupled reaction–diffusion Equation 1:

$$\begin{array}{l} {\left[\mathrm{Ca}\right]}_{t}=D_{\mathrm{Ca}}({\left[\mathrm{Ca}\right]}_{\mathrm{rr}}+\frac{n-1}{r}{\left[\mathrm{Ca}\right]}_{r})\\ -k_{\mathrm{on}}[\mathrm{Ca}][F]+k_{\mathrm{off}}[\mathrm{CaB}] \end{array}$$ (1)

$${\left[F\right]}_{t}=D_{\text{buffer}}({\left[F\right]}_{\mathrm{rr}}+\frac{n-1}{r}{\left[F\right]}_{r})-k_{\mathrm{on}}[\mathrm{Ca}][F]+k_{\mathrm{off}}[\mathrm{CaB}]$$

Governing free Ca^2+^, free buffer (*F*), and Ca^2+^-bound buffer (*B*) concentrations. Here, the Laplacian is written in *n*-dimensional form (Carslaw and Jaeger, 1986; Crank, 1979). The model incorporates diffusion coefficients and reversible binding kinetics characterized by association (*kon*) and dissociation (*koff*) rate constants representative of cytoplasmic Ca^2+^-binding proteins. The Ca^2+^-buffering constants (Table 1) have typical values for cytoplasmic calcium-binding proteins: *kon* = 3^.^108 M^−1^ s^−1^ corresponds to diffusion-limited binding, *Bo* = 0.2 mM, and *DCa* = 220 μm^2^/s. Numerical values for the model parameters are summarized in Table 1.

**Table 1:** Basic definitions and model parameters.

Abbreviation	Definition	Numerical value
*B_o_*	Total buffer concentration	0.2 mM
*c =* [Ca^2+^]*/B_o_*	Normalized Ca^2+^ concentration	0–1
*f =* [*F*]*/B_o_*	Normalized free buffer concentration	0–1
*b =* [*B*]*/B_o_ = 1 - f*	Normalized Ca^2+^-bound buffer concentration	0–1
*D_Ca_*	Ca^2+^ diffusion coefficient	220 μm^2^/s
*d = D_buffer_/D_Ca_*	Relative buffer diffusion coefficient	0.03–1
*k_on_*	On-rate binding constant	3 × 10^8^ M^−1^ s^−1^
*k_off_*	Off-rate constant	10^2^ s^−1^
*K_d_*	Ca^2+^-buffer dissociation constant	0.3 μM
[Ca^2+^]*_o_*	Ca^2+^ concentration at the source	2–200 μM
*τ = 1/k_on_B_o_*	Intrinsic time constant	50 μs
*r_o_ = √D_Ca_/B_o_k_on_*	Intrinsic space constant	105 nm

Analytical solutions were derived using the LG/WKBJ formalism (Sections 5.1 and 5.2). To leading order, the resulting expressions provide compact closed-form approximations for free Ca^2+^ and bound buffer distributions in both one-dimensional and multidimensional geometries. For the 1D case:

$$[Ca^{2+}]=A\frac{\exp(-\mathrm{zQ}-z^{2}/2)}{\surd (z+Q)}$$ (2)

Where A is the concentration of Ca^2+^ at the boundary; z = x/2√t is a self-similarity (Boltzmann) variable, *Q = √*(*z*2 + 1), and *D = √*(*1 - d*)/2, with the normalized parameter *d = Dbuffer/DCa*. The concentration of free buffer is defined by

$$[F]=B_{o}\frac{K}{\left([{\mathrm{Ca}}^{2+}]+K\right)}\;$$ (3)

And:

$$[\mathrm{CaB}\;]=B_{o}\frac{\left[\mathrm{Ca}\;\right]}{\left([\mathrm{Ca}\;]+K\right)}$$ (4)

Where the non-dimensional dissociation constant *K = Kd/Bd=* 0.003 (Table 1). The expressions are consistent with the RBA and Michaelis–Menten formalism. For radial diffusion (the 3D case), all concentrations are simply divided by *r*; for the 2D case, a numerical solution for free Ca^2+^ is preferable because the analytical formulas are cumbersome. The data in Section 5.3 show that Equations 2–4 reproduce well results obtained by direct numerical solution of RD-PDE reaction–diffusion equations.

## 3 Results

### 3.1 Theoretical prediction of one- and two-dimensional Ca^2+^ profiles

Fluorescence measurements report Ca^2+^ bound to the indicator rather than free Ca^2+^ itself, and the theoretical analysis therefore focuses on the dynamics of the bound species. The LG/WKBJ formalism was used to derive analytical expressions for the spatiotemporal distributions of free Ca^2+^ and Ca^2+^-bound buffer species in Section 5.2. Figure 1 compares solutions obtained for slowly diffusing buffers (relative diffusion coefficient *d* = *Dbuffer//DCa =* 0.03) and more rapidly diffusing buffers (*d* = 0.8; e.g., *DFluo−4* = 180 μm2/s, Mironova and Mironov, 2008). In both cases, the leading-order WKBJ approximation closely matches the solution including the first-order correction, demonstrating rapid convergence of the expansion and indicating that the leading term alone can provide a good approximation.

**Figure 1:**
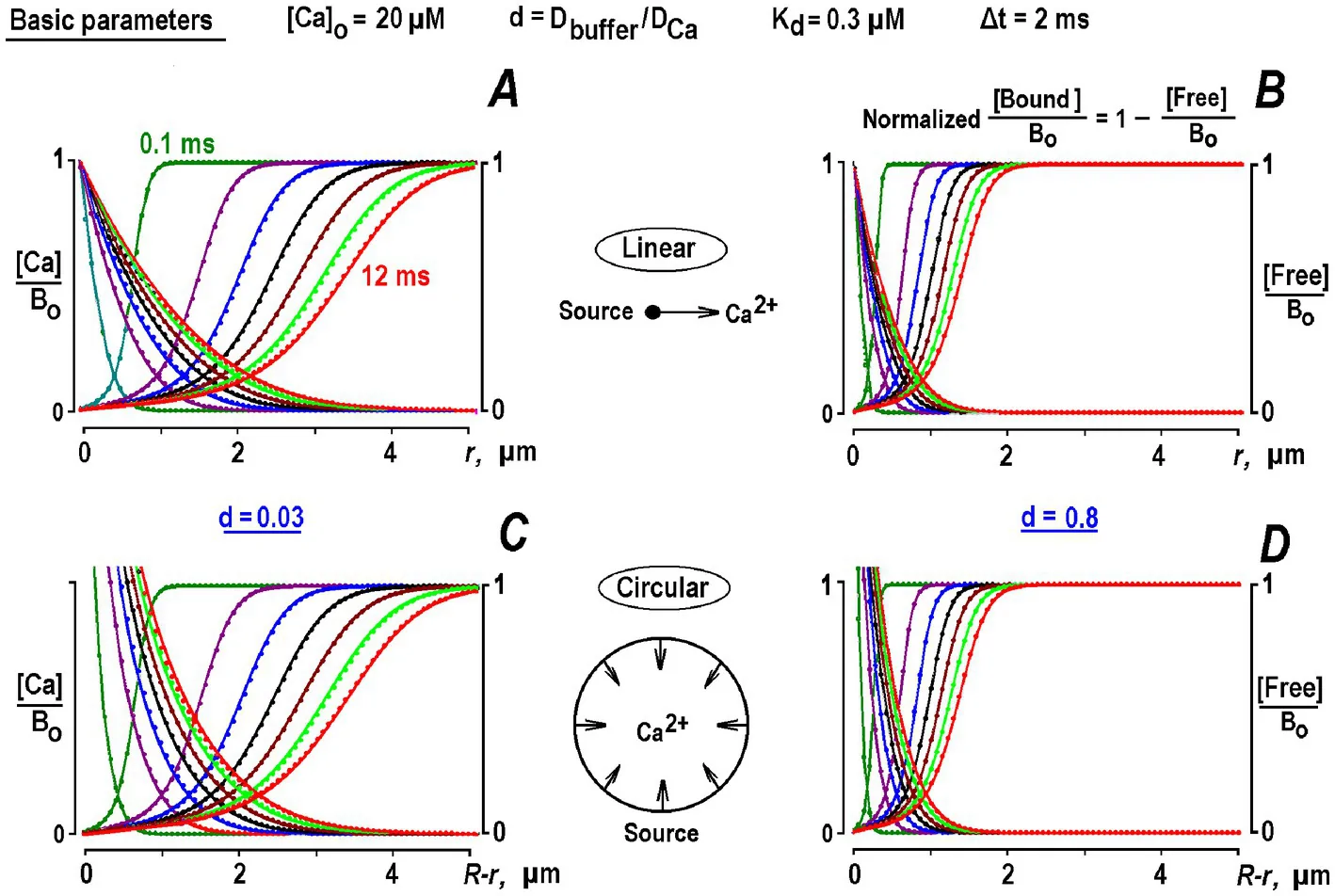
Time- and spatial dependencies of free Ca2+ and buffer. **(A, B)** Linear diffusion from the point source. **(C, D)** Diffusion from the surface surrounding a circular cell. Differently colored curves are placed 2 ms apart and start from 0.1 ms. They present a leading WKBJ term solution derived in Section 5.2. The dots indicate its correction in the first WKBJ order and indicate marginal contributions. Time and space scales are the same in all panels as well as in Figure 2. In linear case the distance *r* is measured from Ca2+ source and in circular case the distance *R* - *r* is measured from the cell surface (*R* = 6 µm). The concentrations of free Ca2+ and buffer are normalized to the total buffer concentration, *B*_o_ = 0.2 mM. Left panels **(A, C)** present solutions for basic model parameters (Table 1, also enlisted in top string). In right panels **(B, D)** the relative diffusion coefficient *d* increased from 0.03 to 0.8 as indicated. Free Ca2+ show a steady increase that slowly depletes buffer at larger distances. Because a sum of normalized [bound Ca2+] and [free buffer] is equal to 1, they mirror each other and the concentration of bound Ca2+ can be readily obtained. Note that in both 1D- and 2D-cases the patterns of free Ca2+ and buffer become close at big distances from Ca2+ source. For circular geometry they deviate only at small distances due to effect of the term c_r_/r in the 2D-Laplacian and correspondingly cut off from above in the panels **C** and **D**. The data are generated for a constant Ca2+ concentration at the source.

For slowly diffusing buffers, free Ca^2+^ spreads progressively from the source as the buffer becomes locally depleted. In contrast, when buffer mobility is closer to that of free Ca^2+^, both free and bound Ca^2+^ distributions remain substantially more confined. This behavior is consistent with the narrow Ca^2+^ nanodomains typically inferred from measurements obtained with small organic fluorescent indicators, whose diffusion coefficients are only moderately lower than that of free Ca^2+^. Comparison of one-dimensional and circular geometries shows that the predicted free Ca^2+^ profiles resemble each other at distances greater than 1 μm and times exceeding 2 ms. Deviations near the source arise from the geometry-dependent Laplacian term and become negligible at larger distances.

For a sustained Ca^2+^ source, free Ca^2+^ concentrations increase continuously because progressive buffer saturation reduces local buffering capacity. In contrast, the distributions of Ca^2+^-bound and free buffer evolve as traveling fronts that propagate away from the source. Calculated velocities are similar for linear and circular geometries (Table 2), indicating that this behavior is largely independent of spatial dimensionality.

**Table 2:** Estimated velocities (μm/s) of Ca^2+^-bound buffer waves.

Theory	Default	d = 0.8	[Ca^2+^]_o_/*B_o_* = 2 μM	= 200 μM	*K_d_* = 0.1 μM	= 1 μM
Linear	325	120	141	322	304	145
Circular	340	128	152	335	321	150
*Experiment*	1D:	α-Syn	2D-DRG:	Control	Bay K 8044	Nifedipine
Fluo-4		191 ± 22		155 ± 34	192 ± 37	114 ± 11
Fluo-4-dex		421 ± 21		382 ± 27	477 ± 38	142 ± 17

Theoretical velocities from front position at 12 ms from data shown in Figures 2, 3. In the second column the numbers are obtained for basic (default) model parameters. In next columns the velocities are obtained in calculation runs, when of only one parameter in the basic set was modified as indicated in the first line. Experimental velocity estimates give the mean wave speeds ± SD as measured for Ca2+-bound buffer (organic indicator dye, fluo-4(X)) in 1D- (single α-Syn channel, Figure 5) and 2D-systems (DRG neurons, Figure 6). Experimental estimates were obtained from FWHM (midpoints) of linescans in Figures 5, 6 divided by elapsed time. The data were analyzed for four different patches with α-Syn channels and four whole-cell patched cells. Control values vs. BAYK data had *P* values equal to 0.0006 (Fluo-4) and 0.0088 (Fluo-4-dextran) respectively.

The calculations shown in Figure 1 assume sustained Ca^2+^ flux. For channels with shorter open times, such as voltage-gated Ca^2+^ channels, the expansion of profiles would be terminated upon channel closure and therefore acquire spatially narrower patterns. Under these conditions, the apparent width of the free Ca^2+^ domain is determined jointly by diffusion and channel open time, whereas the distribution of bound Ca^2+^ additionally reflects the kinetics of buffer binding. Consequently, the duration of Ca^2+^ influx may critically influence downstream signaling by determining whether sufficient sensor activation can occur before channel closure.

One possible test of the proposed model would involve examining the correlation between the opening duration of Ca^2+^ channels and the activity of Ca^2+^-dependent channels when they are present in the same patch. A previous study (Mironov, 2015) found that synuclein channel activity did not alter the activity of delayed rectifier channels (Kdr) but modulated ATP-sensitive K^+^ (KATP) channel activity through changes in submembrane ion homeostasis. It would be interesting to correlate the opening of Ca^2+^ channels and Ca^2+^-activated K^+^ (KCa) channels. However, a preparation in which KCa channels are abundant would be required; this is not the case for the hippocampal neurons used in this study. The long-term effects of bound Ca²+ could be expected for non-selective Ca²+-permeable channels as NMDA and transient receptor potential (TRP) channels (Mironov, 2008).

Figure 1 illustrates the effects of key model parameters. After normalization to the Ca^2+^ concentration at the source, the shape of the free Ca^2+^ profile is largely independent of influx amplitude. In contrast, increasing the source Ca^2+^ concentration markedly accelerates propagation of the bound species while preserving similar wave morphology in both one- and two-dimensional geometries. This behavior arises because elevated Ca^2+^ rapidly saturates local buffer molecules, causing the depletion front to propagate outward.

**Figure 2:**
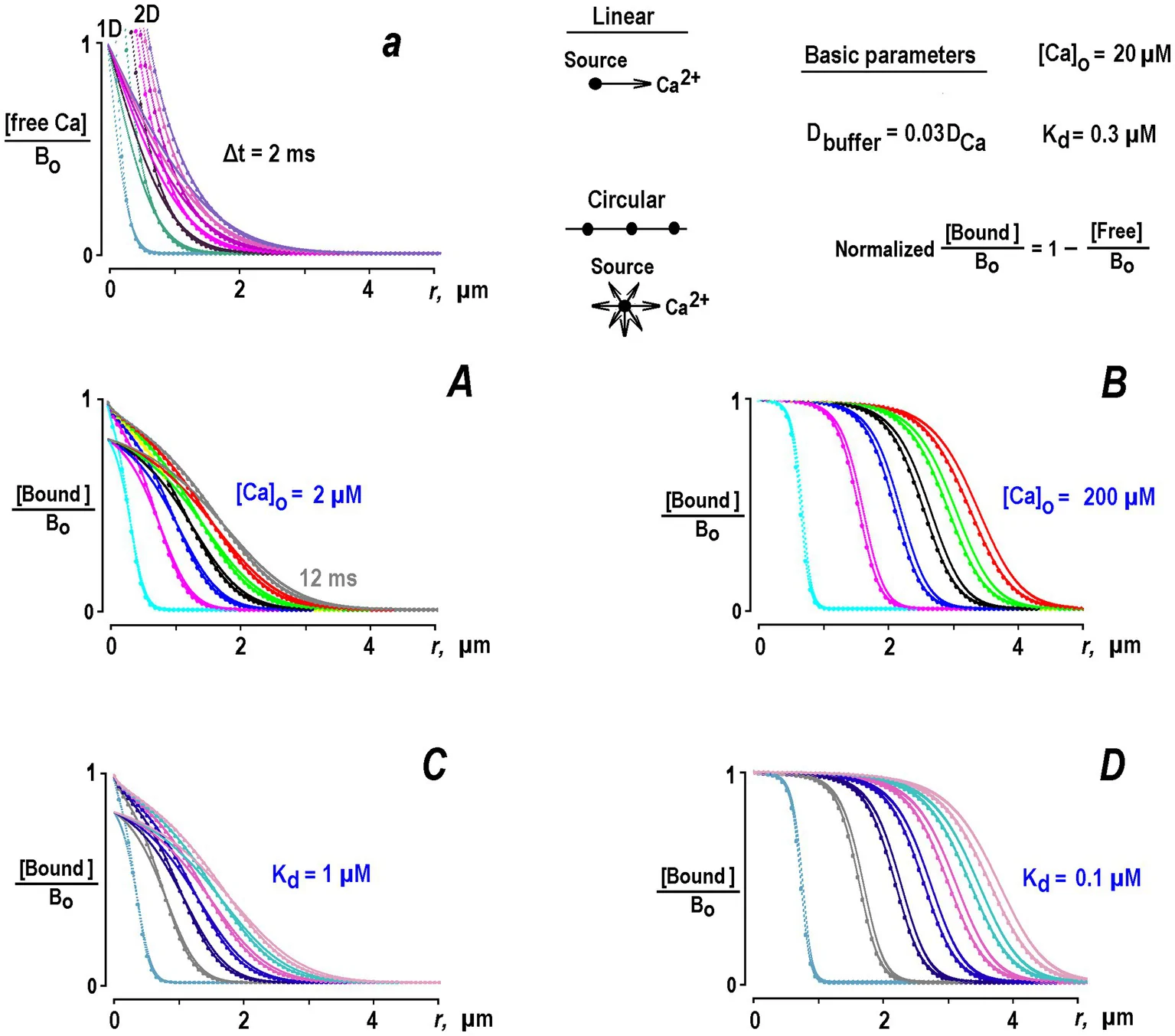
Calculated Ca2+ profiles for different model parameters. The data present leading term WKBJ solution derived in Section 5.2. The notations are the same as in Figure 1. Only results for a slowly moving buffer (*d* = 0.03) are presented. ‘Default’ model parameters (Table 1), are shown also in right top corner. In each calculation, only one key parameter, [Ca2+]_o_ or *K*_d_, is modified. Individual curves start from 0.1 ms and plotted at 2 ms time interval. Free Ca2+ diffused from the point source (inset in the middle) and 1D- and 2D-profiles are shown only once in top left **(a)**. In all cases presented they scaled proportionally according to [Ca2+]_o_ at the source. Initial free Ca2+ is much bigger in 2D-geometry. At distances >1 µm, and times >2 ms, the results for 1D-(lines) and 2D-geometries (lines with circles) practically coalesce. For other parameters, the profiles for Ca2+-bound buffer changed: for small [Ca2+]_o_ = 2 μM **(A)**, bound Ca2+ at big distances follows expansion of free Ca2+**(a)**, but showed slightly slower decay. At bigger [Ca2+]_o_= 200 μM, the pattern of bound Ca2+ is more extended, keep on increasing after free Ca2+ long subsides **(B)**. For *K*_d_ = 0.1 μM **(D)**, bound [Ca2+] propagates faster, whereas for bigger dissociation constant (*Kd* = 1 μM, **(C)**, the profiles of bound [Ca2+] are more dense, resembling those obtained with small [Ca2+]_o_ = 2 μM **(B)**.

The dissociation constant likewise influences wave propagation. Lower *K_d_* values produce faster-moving fronts, whereas larger *K_d_* values yield slower and more spatially confined propagation owing to more rapid equilibration between free and bound states. However, across all parameter combinations examined, the qualitative propagating behavior of Ca^2+^-bound species remains robust.

The sigmoidal shape of the propagating fronts follows directly from the analytical solution. Under the rapid-buffer approximation, the concentration of bound buffer can be expressed as a Michaelis–Menten-type function of free Ca^2+^, Equation 4. The resulting bound fraction naturally assumes a hyperbolic tangent–like profile, with its midpoint determined primarily by the effective dissociation constant. Under the RBA (Michaelis and Menten, 1913) approximation, the concentration of Ca^2+^-bound buffer can be expressed as:

$$\frac{\left[\mathrm{CaB}\right]}{B_{o}}=\frac{e^{\left(-s-\mathrm{lnK}\right)}}{\left(e^{\left(-s-\mathrm{lnK}\right)}+1\right)}=[\tanh((s+\mathrm{lnK})/2)+1]$$ (5)

Thus, the logarithm of the dissociation constant serves as the midpoint of the wave of bound Ca^2+^. When K increases, its logarithm becomes more negative, which leads to denser spacing of the profiles (e.g. Figure 2C vs. Figure 2D).

### 3.2 One-dimensional propagation of Ca^2+^-bound species generated by single α-synuclein channels

To test the theoretical predictions experimentally, one-dimensional Ca^2+^ profiles were generated using outward currents through individual α-synuclein (α-Syn) channels incorporated into excised inside-out membrane patches. The channels are approximately equally permeable to K^+^, Na^+^, and Ca^2+^ (Mironov, 2015), but because the bath contains excess Ca^2+^, the recorded ‘outward’ current represents an almost pure Ca^2+^ current through a single α-Syn channel. This preparation offers the advantage that channel openings frequently persist for several milliseconds, permitting direct observation of the temporal evolution of Ca^2+^-dependent fluorescence signals.

The patches were held at a potential that generated a pure 0.2-pA Ca^2+^ current, matching a typical single L-type channel current (Mironov and Richter, 1998) and corresponding to [Ca2^+^]_o_/*B*o = 0.1 for the default parameter set (Table 1). Figure 4 shows that opening of a single channel produced localized increases in Fluo-4 or Fluo-4-dextran fluorescence corresponding to the formation of Ca^2+^-bound indicator molecules. Kymographs revealed a progressive expansion of the fluorescence signal from the pipette tip toward the pipette interior. Line scans extracted at successive time points demonstrated that the fluorescence front propagated continuously away from the source rather than remaining spatially stationary.

The observed propagation is consistent with the predicted wave behavior of Ca^2+^-bound species. Although fluorescence decayed rapidly, following channel closure because of diffusion and ongoing binding reactions, the spatial evolution during channel opening was well described by a propagating front. Mean experimental velocities are summarized in Table 2, and these velocities agree qualitatively with theoretical predictions.

### 3.3 Two-dimensional profiles of bound Ca^2+^ in the neuronal soma

The theoretical predictions were further evaluated in a two-dimensional cellular geometry using cultured dorsal root ganglion neurons loaded intracellularly with Fluo-4 or Fluo-4-dextran. Whole-cell Ca^2+^ currents were evoked by depolarizing voltage steps, producing reproducible increases in intracellular fluorescence.

At the beginning of the experiment, the voltage pulses produced an increase in the middle of the cell due to excitation of the basal membrane. When the cell was ‘trained’ with 100-ms-long pulses delivered at 0.1 Hz to deplete interstitial space, this offset was markedly reduced, and the lateral transients originating at the cell borders were seen more clearly. Immediately after cell excitation, fluorescence first increased beneath the plasma membrane before progressively spreading into the bulk cytoplasm. Line-scan analysis demonstrated that the resulting Ca^2+^-bound fluorescence propagated inward as a traveling front (Figures 3,4).

**Figure 3:**
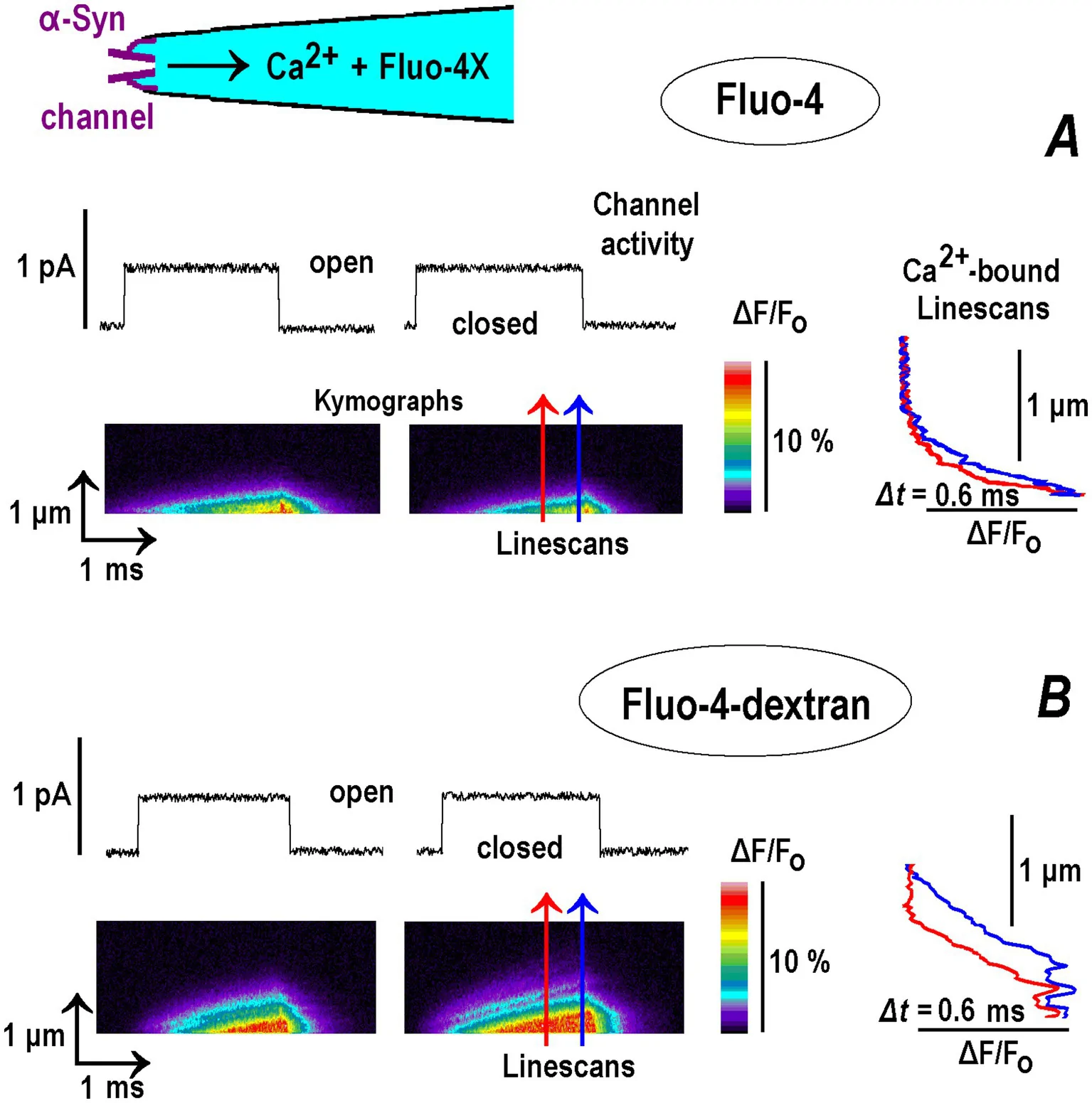
1D-Ca2+transients generated by α-synuclein (α-Syn) channel. Single channels were incorporated into inside-out patches excised from cultured hippocampal neurons. The pipette solution contained either fluo-4 **(A)** or fluo-4-dextran **(B)**. The experimental configuration is sketched in the inset (top left). Channel opening induced ‘outward’ Ca2+ current (the bath contained 88 mM CaCl2) and related local increases of Ca2+ bound to the indicator dye in the pipette tip. The panels show sample events obtained in four patches for each indicator dye. Note that after channel closure, the fluorescence decay fast due to diffusion of Ca2+ and reaction with free buffer molecules in the bulk. The panels **A** and **B** show a single channel current (top) and the fluorescence of bound Ca2+ as kymographs (bottom) The relative changes in fluorescence are obtained from background subtracted images and plotted according to the color calibration bar in the middle. The time runs from left to right and the distance from the pipette tips runs upward. Increasing normalized fluorescence (*F/F*o) corresponds to an increase in bound Ca2+ (warmer colors). A monotonous increase in fluorescence indicates an expansion of bound Ca2+. Rightmost in both panels shown are the distance scans made as indicated by arrows of same color showing also the direction of scanning.

**Figure 4:**
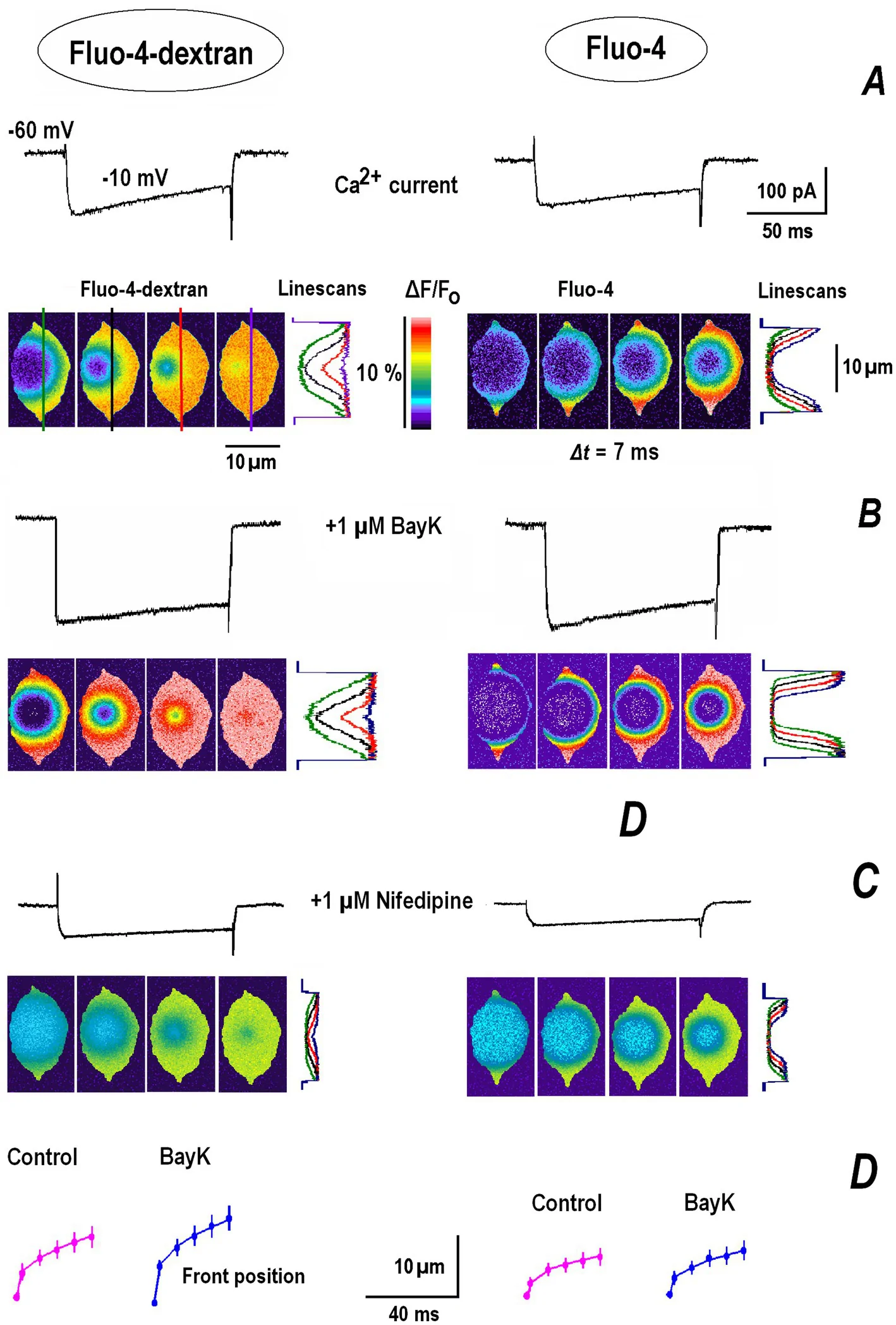
2D-'circular' Ca^2+^ diffusion in dorsal root ganglion (DRG) neurons. Each panel shows whole-cell Ca^2+^-channel current evoked by step depolarizations by 100 ms-long pulse from -60 to -10 mV (top traces). The cell images (taken at interval 7 ms starting from 1.5 ms) were used to obtain the vertical linescans through the middle of images indicated as vertical lines with the same color in **(A)**. The panels show the results obtained in the control **(A)**, 5 min after addition of agonist of L-type Ca^2+^-channels **(B)** and its wash-out with antagonist **(C)**. The last panel **D** shows the plots of measured front positions vs. time as means ± SD obtained from four cells for each indicator dye. The lines depict appropriately scaled √t-dependence indicative a diffusion process.

Pharmacological manipulation of Ca^2+^ influx produced the changes predicted by the analytical model. Enhancement of L-type Ca^2+^ channel activity with Bay K 8644 increased current amplitude and accelerated propagation of the fluorescence front, whereas inhibition with nifedipine reduced both current amplitude and wave velocity. These effects were observed for both indicator dyes but were particularly pronounced with the slowly diffusing dextran-conjugated probe. Front position changed in proportion to √t, which is characteristic of a diffusion process (the self-similarity variable is *z = x/2√t*; see Equation 2 above and Section 5.1.).

The experimentally determined propagation velocities (Table 2) followed the theoretical trends predicted by the WKBJ analysis: larger Ca^2+^ influxes produced faster-moving waves, whereas reduced influx slowed propagation. Moreover, the overall similarity between the one-dimensional and two-dimensional measurements supports the conclusion that the spread of Ca^2+^-bound species represents a robust consequence of RD dynamics rather than a feature specific to a particular experimental geometry.

## 4 Discussion

The present study complements and extends two widely used concepts in biochemistry and biophysics: the Michaelis and Menten (1913) formalism and the Excess Buffer Approximation (EBA) introduced by Neher (1986). The Michaelis–Menten approach describes local substrate–enzyme interactions but does not account for the diffusion of either the substrate or the enzyme. Similarly, the EBA assumes that Ca^2+^ buffers are present in excess and therefore remain far from saturation. This assumption permits linearization of the original RD system (Equation 1), reducing it to second-order ordinary differential equation. The resulting solution predicts the formation of localized Ca^2+^ nanodomains around persistently open Ca^2+^ channels. This concept is frequently invoked to explain a variety of physiological phenomena, as recently reviewed by Eisner et al. (2023).

The majority of theoretical studies of intracellular Ca^2+^ signaling have focused on exponentially decaying steady-state Ca^2+^ distributions (Neher, 1986; Naraghi and Neher, 1997; Smith et al., 2001). However, these analyses critically depend on the assumption of excess buffer. When this constraint is removed, the analytical solution for irreversible Ca^2+^ binding with nearly equal Ca^2+^ and buffer diffusion coefficients predicts the appearance of periodic Ca^2+^ patterns under specific conditions (Mironov, 2019). Exact solutions were also found for an immobile buffer that binds Ca^2+^ irreversibly (Mironov, 2023a; the RBA does not apply in this case). An important conclusion emerging from these analyses is that free Ca^2+^ and Ca^2+^-bound species may exhibit markedly different dynamic behaviors. While free Ca^2+^ accumulates relatively slowly around an individual channel, Ca^2+^ bound to a buffer or sensor can propagate away from the source at substantially higher velocities (Mironov, 2023a). Consequently, the effective signal carriers—the Ca^2+^-bound buffer or sensor molecules—may travel both faster and farther than the signaling ion itself.

To fully understand the information conveyed by intracellular Ca^2+^ signals, it is necessary to consider not only the distribution of free Ca^2+^ but also the generation and propagation of Ca^2+^-bound buffers and sensors. Fluorescence measurements report Ca^2+^ bound to the indicator rather than free Ca^2+^ itself, and the theoretical analysis therefore focused on the dynamics of the bound species. In the theoretical part of this study, we used the RBA, which is identical to Michaelis–Menten kinetics widely used in biochemistry. Wagner and Keizer (1994) were the first to apply the RBA to intracellular Ca^2+^ buffering and derived a non-linear RD equation for total cellular Ca^2+^. Their analysis relies primarily on numerical solutions and limiting cases. However, approximate analytical solutions are possible under the RBA when a logarithmic approximation of total Ca^2+^ is used (Mironova and Mironov, 2008).

The present study further extends this concept by explicitly incorporating diffusion into Michaelis–Menten-type reaction kinetics. To derive analytical solutions, the LG transformation was used, as described in Sections 5.1 and 5.2. In quantum mechanics, this approach is widely known as the WKBJ method and is commonly used to solve the stationary Schrödinger equation. Applications of WKBJ techniques to time-dependent RD systems are comparatively rare. Among the few examples are the studies by Krause et al. (2020), although these authors used a stationary WKBJ formalism in which temporal dependence entered only indirectly.

To avoid overburdening the main text, all mathematical derivations and numerical analyses are presented in Sections 5.1 and 5.2. As an initial validation of the approach, the WKBJ formalism was applied to multidimensional diffusion and to diffusion coupled with a first-order reaction corresponding to the time-dependent version of the EBA (Section 5.1). The resulting solutions accurately reproduced established analytical results. Remarkably, the leading-order WKBJ approximation alone proved sufficient to achieve excellent agreement with exact solutions (Section 5.1; Figure 5).

**Figure 5:**
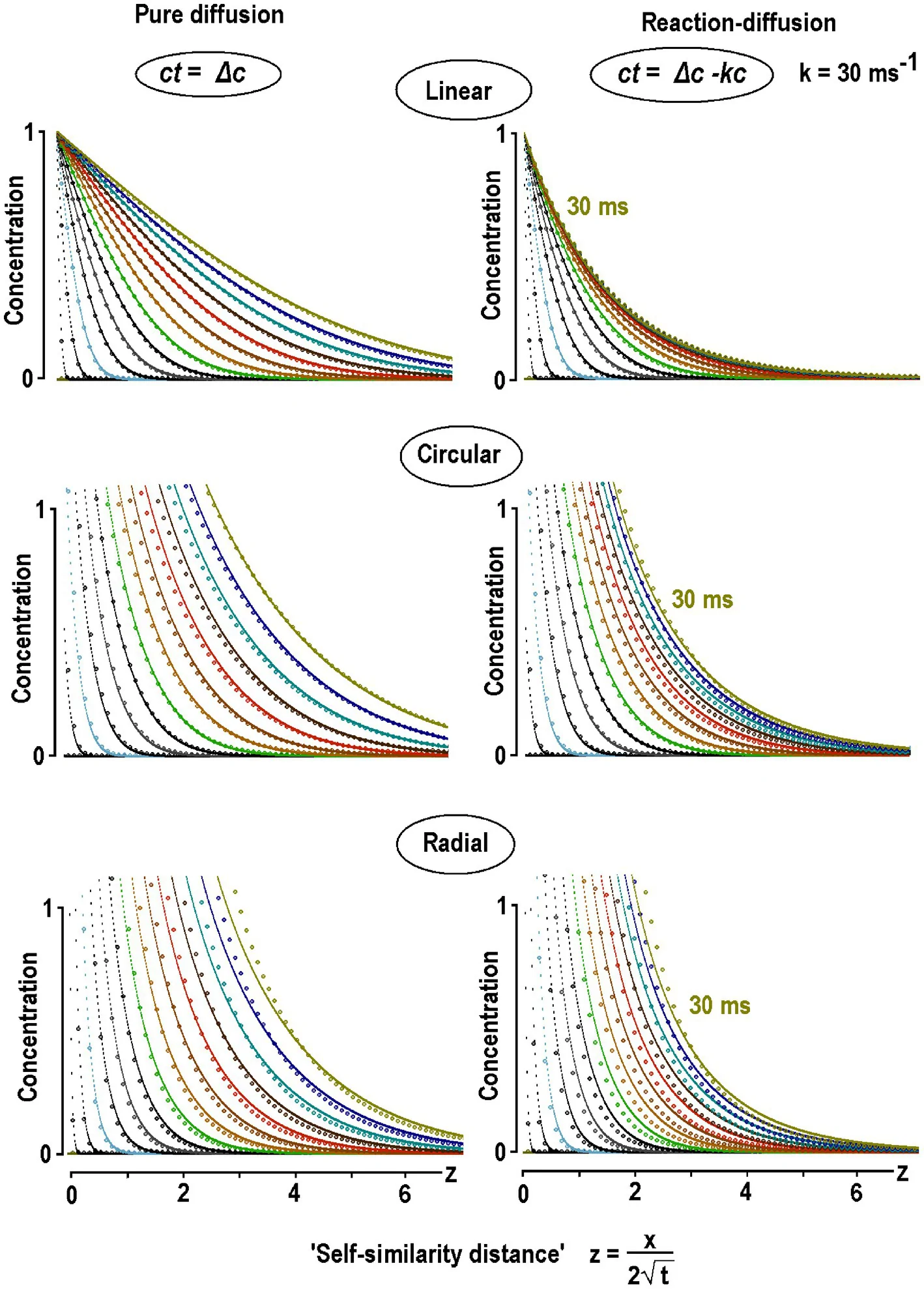
LG-WKBJ approach to solve reaction-diffusion PDEs. Solutions are obtained for multi-dimensional (1-, 2- and 3-D) diffusion (left panels) and reaction-diffusion equations (right panels) as indicated. Exact solution of diffusion (*ct* = Δc) and reaction-diffusion equations (*ct* = Δ*c*-*kc*, *k* = 30 ms^-1^) are plotted as lines. Time-dependent changes given by established analytical solutions (lines) are well reproduced by the leading term in LG-WKBJ (dots). For linear and radial geometries the curves are calculated from explicit expressions listed in Table 3, and for circular diffusion they are obtained by numerical integration of Equation A5. Concentrations (*y*-axes) are plotted vs. the Boltzmann variable (a self-similarity ‘distance’). In 30 ms-long runs, the time interval between the curves increased quadratically to obtain about equally separated curves. The profiles become sharper with increasing dimensionality, and the curves obtained in 2D- and 3D geometries are cut from above. Introduction of ‘diffusion sink’, which describes the bulk uptake with the rate constant *k*, decreases the width of profiles that ‘saturate’ with time (right row of panels).

The same methodology was subsequently extended to the Ca^2+^–buffer system (Section 5.2). The resulting derivations yield simple and transparent analytical expressions, as summarized in Equations 2–4. Once again, the leading-order WKBJ term appeared sufficient to reproduce the behavior of multidimensional RD systems (Figure 1). Although only a single buffer species was considered here, the formalism can readily be extended to multiple buffers with different mobilities, as previously proposed for related RBA models (Mironova and Mironov, 2008).

The theoretical analysis identifies buffer mobility as a major determinant of intracellular Ca^2+^ patterns (Figure 1). Contrary to the predictions of the classical EBA, these patterns are not stationary, even under conditions of constant Ca^2+^ influx. Owing to progressive buffer saturation, free Ca^2+^ distributions continuously expand away from the channel source in both linear (Figures 1A,B) and circular (Figures 1C,D) geometries. Even more striking behavior is observed for Ca^2+^-bound species, whose distributions spread over substantially larger distances.

For buffer diffusion coefficients approaching that of free Ca^2+^ (Figure 1B), the distributions of both free and bound Ca^2+^ remain spatially confined, thereby supporting the classical concept of Ca^2+^ nanodomains (Neher, 1986). This paradigm was used to interpret measurements performed using synthetic fluorescent indicator dyes whose diffusion coefficients are close to that of free Ca^2+^. Endogenous cytoplasmic Ca^2+^ buffers such as calmodulin are larger molecules and possess substantially lower diffusion coefficients; consequently, they are often treated as effectively immobile buffers (Zhou and Neher, 1993; Schwaller, 2010). Both the theoretical predictions presented here (Figures 1, 2) and the corresponding experimental observations suggest that, under conditions of low buffer mobility, Ca^2+^-bound buffer and sensor molecules are not confined to localized domains but instead propagate progressively throughout the cytoplasm.

## 5 A novel mathematical treatment of RD systems using the WKBJ formalism

### 5.1 Time-dependent solutions of simple diffusion equations

Before applying the LG/WKBJ formalism to Ca^2+^ buffering, we first validate the approach using diffusion and RD equations for which exact analytical solutions are available. This comparison demonstrates that the leading-order WKBJ approximation accurately reproduces the known analytical solutions and provides the foundation for the subsequent analysis.

Consider a non-stationary (time-dependent) case of simple diffusion:

$$c_{t}=\mathrm{\Delta c}$$ (A1)

Where subscripts *t* and *r* denote the time and spatial derivatives, respectively, and *Δ* represents the *n*-dimensional Laplacian:

$$\mathrm{\Delta c}=c_{\mathrm{rr}}+\frac{n-1}{r}c_{r}$$

Used in Equation 1 (Crank, 1979; Carslaw and Jaeger, 1986). A straightforward way to obtaining the solution is to introduce the Boltzmann (Crank, 1979) or self-similarity variable *z = r/2√t* (Polyanin and Zaitsev, 2004). Equation A1 then transforms into the following ordinary differential equation (ODE):

$$c^{\prime \prime }+(2z+\frac{n-1}{z}c^{\prime })=0$$ (A2)

Where primes indicate a derivative with respect to z. For further analysis, the equation is transformed into a canonical (LG) form through a change of variable (Polyanin and Zaitsev, 2004):

$$c=u\;\exp(-\int_{0}^{z}[z+\frac{n-1}{z}]\mathrm{dz})$$

That gives:

$$u^{\prime \prime }-Q(z)u=0$$ (A3)

Where:

$$Q(z)={\left(2z+\frac{\left(n-1\right)}{z^{2}}\;\right)}^{2}/4+(2-\frac{\left(n-1\right)}{z^{2}}\;)/2$$

Equation A3 is simply a stationary Schrödinger equation with an effective potential *Q*(*z*). Following Bender and Orszag (1999), we rewrite it as.

$$h^{2}u^{”}-Q(z)\;u=0$$ (A4)

Where *h* is a small parameter. The dependent variable is then presented as an exponential, whose argument is expanded in powers of *h* as

$$u=\exp(\frac{s+\mathrm{hS}+\ldots }{h})$$ (A5)

Next, substitute (Equation A5) into (Equation A4), divide out the exponential factors, and collect the terms multiplied by the same power of *h.* The first two equations have solutions (Bender and Orszag, 1999):

$$\;s=\pm \int_{0}^{z}\surd Q\mathrm{dz}$$ (A6a)

S=−ln(Q)/4 (A6b)

Where s and S are the first and second terms in the WKBJ expansions, respectively (Equation A8). In most WKBJ applications, further correction terms are small, and only two terms in the expansion of the exponent are sufficient for most purposes (Bender and Orszag, 1999). This also applies in the present study, as shown below. For example, when Q(z) = *k*, the solution given by Equation A6a,

$$S=\exp(-\sqrt{\mathrm{kx}})$$

Is identical to the analytical solution of ODE *c” = kc*, which appears as a steady-state solution under the EBA (Neher, 1986).

For a physically relevant solution, only the exponential with a negative argument (minus sign) in (Equation A6a) is used. For multidimensional diffusion, the equations, potentials Q(z), and leading solutions *lnc* = s(*z*) are given in Table 3. In the 1D case, the integral (Equation A6a) is

$$\int_{0}^{z}\surd Q\mathrm{dz}=[\mathrm{zQ}/2+\ln(\frac{z}{Q}+1)/2]$$ (A7)

**Table 3:** Explicit expressions of functions used in derivation of leading term in WKBJ solutions for multi-dimensional diffusion.

Expression	Linear	Circular	Radial
(A3)	*Δc=crr*	*Δc=crr + crr/r*	*Δc=crr + 2crr/r*
(A6) Q(z)	z^2^+1	z^2^+2-1/4z^2^	z^2^+3
(A10) lnc = s(z)	−[zQ+ln(z/Q+1)]/2	−∫√Q(z) dz	−[zQ+ln(z/Q+3)]/2
Exact	erfc(z)*	Cumbersome	erfc(z)/r

* $\mathrm{erfc}(z)=\int_{z}^{\infty }\exp(-z^{2})\mathrm{dz}$ , a complementary error function.

For circular diffusion, direct integration of the polynomial *Q*(*z*) = √(z2 + 2–1/4z2) is possible, but the result is cumbersome and involves elliptic integrals. Therefore, the original Equation A6 was instead numerically integrated by the method of lines (Berezin and Zhidkov, 1965; Mironov, 2023a), as briefly described in Section 5.3. In the 3D case, the solution is simply the 1D result divided by *r*.

Figure 4 shows that the leading WKBJ term (Equation A6a) closely reproduces the exact solutions (left panels), and the first-order contribution (Equation A6b) does not modify the results substantially.

Next, consider the simplest RD equation:

$$\;c_{t}=△c-\mathrm{kc}$$ (A8)

Compared with pure diffusion, it contains a spatially uniform first-order reaction, whose rate constant is *k*. Equation A8 corresponds to the time-dependent excess buffer approximation (EBA), which describes irreversible Ca^2+^ binding by the buffer with rate constant *k = k_on_B_o_* (Table 1). After transformation into a self-similar form, the equation corresponds to (Equation A3), with *4ktc* on the right-hand side. The solutions are readily obtained from (Equation A10) in Table 3 with this term added to Q(z), as determined for pure diffusion. The exact solution (Mironov, 2019) is:

$$c=[e^{-x}\mathrm{erfc}(z-\sqrt{t})+e^{+x}\mathrm{erfc}(z+\sqrt{t})]/2$$

This solution is again closely reproduced by the leading term (Figure 5, right panels B, D, and F).

### 5.2 Time-dependent analytical solutions of Ca^2+^ buffering using WKBJ formalism

In all derivations below, we use the normalized equation derived from Equation 1 in Methods. Here, the relative buffer diffusion coefficient is *d = Dbuffer/DCa*, and the intrinsic time and distance units are *τ = 1/konBo* = 50 μs and *ro = √DCa/Bokon* = 105 μm, respectively (Table 1; see also Mironov, 2019, 2023a). The concentrations below are divided by the *total* buffer concentration *Bo* = 0.2 mM (Table 1). The system of RD equations for Ca^2+^ buffering is

$$c_{t}=\mathrm{\Delta c}-\mathrm{cf}+K(1-f)$$ (B1)

$$f_{t}=\mathrm{d\Delta f}-cf+K(1-f)$$

Where *c, f*, and *b* define the concentrations of free Ca^2+^, free buffer, and bound buffer, respectively (all normalized to *Bo*). Note that *b = 1 – f*. The normalized dissociation constant is *Kd = koff/konBo* = 0.003 (Table 1).

The first two terms describe diffusion, and the last two terms represent the forward and backward steps in the reversible reaction. It is convenient to reorganize the reaction term as:

R=−cf+K(1−f)=−(c+K)f+K=−Cf+K

Where a new Ca^2+^-dependent variable *C = c + K* is introduced.

In the ‘multiple-scales’ representation (Bender and Orszag, 1999; see also Equation A4 above), we need to solve the system.

$$hC_{t}=h^{2}△C-\mathrm{Cf}+K/h$$ (B2)

$$hf_{t}=h^{2}\mathrm{d\Delta f}-\mathrm{Cf}+K/h$$

When the reaction term:

$$R=-\exp(\frac{s}{h}+S+\frac{\varphi }{h}+\Phi +\ldots )+\exp(\mathrm{lnK}/h)$$ (B3)

Is expanded in exponential form at leading order *h^−1^*, the reaction term *R = 0*, owing to the identity.

φ+s=lnK (B4)

Or

f=K/(K+c) (B5)

Which describes the equilibrium between Ca^2+^ and the buffer, as presumed in the RBA or Michaelis–Menten formalism.

Expanding the concentrations as exponentials, *C = exp*(s*/h + S +* .) and *f = exp*(*φ/h + Φ + .*), the system (Equation B3) can be written as:

$$hs_{t}=h^{2}△s+hs_{r}^{2}+R_{1}$$ (B6)

$h\varphi_{t}=h^{2}d△\varphi +h\varphi_{r}^{2}+R_{2}$

Where the reaction terms are:

$$R_{1}=-\exp(\varphi /h)+\exp(-s/h+\mathrm{lnK}/h)$$

$$R_{2}=-\exp(s/h)+\exp(-\varphi /h+\mathrm{lnK}/h)$$

It follows from (Equation B4) that *R1 = R2* = 0. Subtracting the two equations in (Equation B6), we obtain a single equation:

$${\left(s-\varphi \right)}_{t}=s_{r}^{2}-d\varphi_{r}^{2}+\mathrm{h\Delta}(s-\mathrm{d\varphi})$$ (B7)

It also follows from (Equation B5) that, for both time and spatial derivatives, *φ’ = −s’*, which simplifies (Equation B8) to:

$$2s_{t}=(1-d)s_{r}^{2}$$ (B8)

This is an equation for pure diffusion with an apparent diffusion coefficient *D =* (*1 - d*)/2. Its solution for different dimensions can be obtained from line Table 3, with *the z* variable scaled by √[(*1 - d*)/2]. Note that the case *d = 1* is critical (see also the end of Section 5.3 below) and should be treated separately using, e.g., the full Equation B1, whose solution was obtained previously (Mironov, 2019).

Subtracting the two equations in (Equation B4) at the next order *h^1^* gives:

$${\left(S-\Phi \right)}_{t}=\Delta (s-\mathrm{d\varphi})+2(s_{r}S_{r}+D\varphi_{r}\Phi_{r})\;$$ (B9)

To express *Φ* through *S*, we write:

$$R=\Phi +S=s\;r2-s_{t}\;$$

From which *Φ* derivatives can be defined as

$$\Phi^{\prime }=R^{\prime }-S^{\prime }\;$$ (B10)

Inserting (Equation B10) into (Equation B9), we obtain a first-order PDE that may be solved analytically (Polyanin et al., 2002), but the explicit solution is rather cumbersome. Therefore, (Equation B9) was solved numerically. The data plotted in Figure 1 in the main text indicate no significant modification of the solution given by the leading WKBJ term (Equation B8).

Explicit formulas for the leading WKBJ term in the 1D formalism are listed in Section 2.6 in Methods, Equations 2–4. The 3D results can be readily obtained from the 1D case by dividing the solution by *r*. The next section and Figure 6 show that the WKBJ ansatz closely reproduces numerical solutions of the full RD system (Equation B1), indicating that the WKBJ approach offers a simple way to incorporate the effects of diffusion into the Michaelis–Menten (or RBA) formalism. The complex is then transformed irreversibly to the product of reaction showing the progression of the reaction.

**Figure 6:**
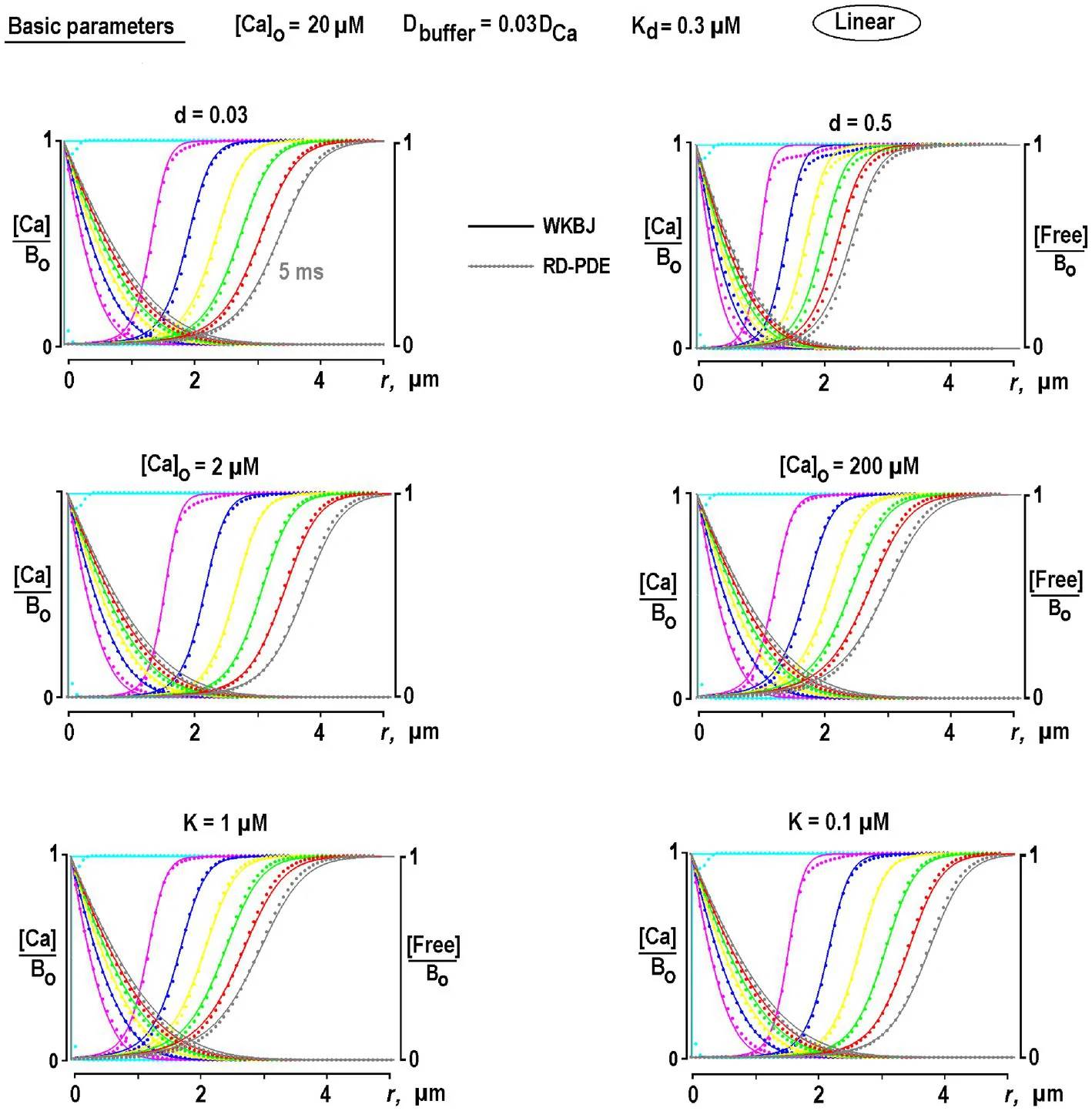
LG-WKBJ ansatz closely reproduces numerical solution of reaction-diffusion PDE. The full RD equation for Ca2+ buffering (Equation B1) was solved by the method of lines (Section 5.3). The lines present the leading term in LG-WKBJ ansatz (lines), and the dots depict numerical solutions of full system (Equation B1). In top left panel the calculations were made with ‘default’ parameters (Table 1) and in other panels only one key parameter was changed as in Figure 2. Only linear case was examined and the results in 2D-geometry show similar trend. The data indicate that LG-WKBJ approach provides a good analytical alternative to numerical approach in solving RD equations.

### 5.3 Application of the method of lines to solving RD equations

The algorithm is illustrated for a typical RD equation.

$$c_{t}=D\;\mathrm{\Delta c}-f(c)$$ (C1)

For diffusion in the presence of a sink, whose concentration dependence may be non-linear. For pure diffusion, the algorithm was described by Berezin and Zhidkov (1965). Below, the algorithm is presented following Mironov (2023b), with appropriate modifications for a non-linear sink. Briefly, write (Equation C1) in discrete form as:

$$D\frac{\;c_{n+2}^{t}-2\;c_{n+1}^{t}+c_{n}^{t}\;\;}{dx^{2}}=[\frac{\;c_{n+1}^{t}-c_{n+1}^{t-\mathrm{\Delta t}}\;\;\;}{\mathrm{dt}}]-f(c_{n+1}^{t-\mathrm{\Delta t}})$$ (C2)

The solution proceeds in two steps. First, we assume a linear relationship between c*tn* and c*tn*_+1_:

$$c_{n}^{t}=C_{n}^{t}\;c_{n+1}^{t}+\phi_{n}^{t}\;$$ (C3)

Setting *γ = D dt/dx^2^*, we present (Equation C2) as:

*γ*(c *^t^_n + 2_ - 2*c *^t^_n + 1_ + c^t^_n_*) *=* (*c^t^_n + 1_ - c^t-Δt^_n + 1_*) - $f(c_{n+1}^{t-\mathrm{\Delta t}})\mathrm{dt}$

As in (Equation C3), we can derive a linear relationship between c*t*n+2 and *ctn+1* as:

$${{c^{t}}_{n}}_{+1}={C^{t}}_{n}{{c^{t}}_{n}}_{+2}+{{\varphi^{t}}_{n}}_{+1}$$ (C4)

Where:

$${{C^{t}}_{n}}_{+1}=1/\mathrm{Den}$$

$$\mathrm{Den}=[2-{C^{t}}_{n}+1/\gamma ]$$

$${{\varphi^{t}}_{n}}_{+1}={\varphi^{t}}_{n}+({{c^{t-\mathrm{\Delta t}}}_{n}}_{+1}-{{f^{t-\Delta }}^{t}}_{n+1}\mathrm{dt})/\mathrm{Den}$$ (C5)

In numerical calculations, the procedure starts from the initial conditions, e.g.:

$$c{‘}_{o}=0,\text{and}\;c_{o}=A$$

And subsequently evaluates all coefficients *C_n_* and *φ^t^_n_* using (Equation C5). The limiting value of *cend* is then set to 0 or another constant and we move backward to obtain all concentrations c_n_ at a given *t* from Equation C3. The two progressions, Equations C4, C5, explicitly define both the forward and backward steps, which explains the name ‘chasing’ for the algorithm (Berezin and Zhidkov, 1965). The algorithm has been tested for many ordinary differential equations (ODEs) and accurately recovered exact solutions (Mironov, 2023b). Compared with other approaches designed to solve RD equations, the main advantage of the method is the ability to use larger time steps without losing accuracy or stability.

The method of lines was used to solve the full Equation B1. Figure 6 presents the results of calculations for the sample cases shown in Figures 1, 2 above. The solutions disagree slightly at *d* > 0.9, close to the critical value *d* = 1. In this case, it is better to solve the full system (Mironov, 2019). Nonetheless, the close agreement between the results obtained analytically and numerically indicates that the LG-WKBJ ansatz may be used as a simple analytical alternative to the numerical solution of RD equations.

## Data Availability

The original contributions presented in the study are included in the article/supplementary material, further inquiries can be directed to the corresponding author/s.
